# Non-replication of an association of *CTNNBL1 *polymorphisms and obesity in a population of Central European ancestry

**DOI:** 10.1186/1471-2350-10-14

**Published:** 2009-02-19

**Authors:** Carla IG Vogel, Brandon Greene, André Scherag, Timo D Müller, Susann Friedel, Harald Grallert, Iris M Heid, Thomas Illig, H-Erich Wichmann, Helmut Schäfer, Johannes Hebebrand, Anke Hinney

**Affiliations:** 1 Department of Child and Adolescent Psychiatry, University of Duisburg-Essen, Essen, Germany; 2 Institute of Medical Biometry and Epidemiology, Philipps-University of Marburg, Marburg, Germany; 3 Institute for Medical Informatics, Biometry and Epidemiology, University of Duisburg-Essen, Essen, Germany; 4 Helmholtz Zentrum München – German Research Center for Environmental Health, Institute of Epidemiology, Munich-Neuherberg, Germany; 5 IBE, Chair of Epidemiology, University of Munich, Munich, Germany

## Abstract

**Background:**

A recent genome-wide association (GWA) study of U.S. Caucasians suggested that eight single nucleotide polymorphisms (SNPs) in *CTNNBL1 *are associated with obesity and increased fat mass. We analysed the respective SNPs in data from our previously published GWA for early onset obesity (case-control design), in GWA data from a population-based cohort of adults, and in an independent family-based obesity study. We investigated whether variants in *CTNNBL1 *(including rs6013029) and in three other genes (*SH3PXD2B*, *SLIT3 *and *FLJ42133*,) were associated with obesity.

**Methods:**

The GWA studies were carried out using Affymetrix^® ^SNP Chips with approximately 500,000 markers each. In the families, SNP rs6013029 was genotyped using the TaqMan^® ^allelic discrimination assay. The German case-control GWA included 487 extremely obese children and adolescents and 442 healthy lean individuals. The adult GWA included 1,644 individuals from a German population-based study (KORA). The 775 independent German families consisted of extremely obese children and adolescents and their parents.

**Results:**

We found no evidence for an association of the reported variants in *CTNNBL1 *with early onset obesity or increased BMI. Further, in our family-based study we found no evidence for over-transmission of the rs6013029 risk-allele T to obese children. Additionally, we found no evidence for an association of *SH3PXD2B*, *SLIT3 and FLJ42133 *variants in our two GWA samples.

**Conclusion:**

We detected no confirmation of the recent association of variants in *CTNNBL1 *with obesity in a population of Central European ancestry.

## Background

Obesity is a major health problem worldwide and results from an interplay of social, environmental and genetic factors [1]. Genome-wide association (GWA) studies have contributed to the identification of new polygenic variants contributing to inter-individual body mass index (BMI) differences [2-5]. Recently, Liu et al. [6] reported that variants in the beta catenin-like 1 gene (*CTNNBL1*) were associated with increased fat mass and obesity in a GWA conducted with 1,000 adult U.S. Caucasians. In the same report, this observation was validated in a French case-control sample (896 class III obese adults; BMI ≥ 40 kg/m^2 ^and 2,916 normal weight controls; BMI < 25 kg/m^2^).

Our study had two objectives. First, we aimed to replicate the association of the obesity risk alleles (rs6013029 T-allele, rs16986921 T-allele, rs6020712 A-allele, rs6020846 G-allele, rs6020395 C-allele, rs16986890 G-allele, rs6096781 C-allele, and rs6020339 C-allele) of *CTNNBL1 *in two GWA data sets. Second, we explored three other genes *SH3PXD2B *(rs13356223, rs10077897 and rs13436547), *SLIT3 *(rs17734503 and rs12654448) and *FLJ42133 *(rs7363432 and rs6095722), also mentioned by Liu et al. [6] in our GWAs. We analysed three samples: (1) GWA data from 487 cases with early onset extreme obesity and 442 controls; (2) GWA data of 1,644 individuals from a population-based adult cohort and (3) genotyping data of the best *CTNNBL1 *SNP rs6013029 previously reported in [6] in a sample of 775 independent nuclear families each comprising one or more extremely obese offspring and both parents.

## Methods

### Participants and Genotyping

#### Case-control GWA

487 extremely obese children and adolescents (cases: mean age 14.38 ± 3.74; BMI = 33.40 ± 6.81; BMI Z-score = 4.63 ± 2.27; 42.9% male) and 442 healthy lean individuals (controls: mean age 26.07 ± 5.79; BMI = 18.31 ± 1.10; BMI Z-score = -1.38 ± 0.35; 38.7% male) from Germany. The use of lean adults who were never overweight or obese during childhood (assessed by interview [7]) as control group reduces the chances of misclassification compared to the use of lean children as controls that might become overweight in adulthood. This sample was genotyped using the Affymetrix^® ^Genome-Wide Human SNP Array 5.0 with 440,794 markers. Details on this GWA have been reported elsewhere [7]. The study was approved by the local ethics committee.

#### Population-based GWA

KORA (Kooperative Gesundheitsforschung im Raum Augsburg, follow up of Survey 3 (F3); '*Cooperative Health Research in the Region of Augsburg*') comprises 3,126 German adults representative of the population within the age range 25–74 years in Augsburg and surrounding areas (Bavaria, Germany). 1,644 probands (mean age 52.52 ± 10.09; BMI = 27.33 ± 4.12; 48.9% male) were genotyped using the Affymetrix^® ^GeneChip^® ^Human Mapping 500K Array Set (for details on the sample see [8]). The ethics committee of the Länderärztekammer for Bavaria approved the study.

#### Family-based study

775 German families comprising 1,058 extremely obese children and adolescents (775 index patients, 283 siblings; mean age 13.88 ± 3.69; BMI 31.12 ± 6.06 kg/m^2^; BMI Z-score = 3.91 ± 2.02, 45.8% male) and 1,550 parents (mean age 42.56 ± 5.95; BMI 30.37 ± 6.29 kg/m^2^; BMI Z-score = 1.68 ± 1.83) were recruited at the University of Marburg and the University Duisburg-Essen. Participants were genotyped for the SNP rs6013029 using the TaqMan^® ^allelic discrimination assay (C_29958195_10 assay, Applied Biosystems, Germany); the call rate was 99.7%, with 100% concordance of duplicates. All individuals studied are Caucasians from Central Europe, with German ancestry. All studies were conducted in accordance with the guidelines of The Declaration of Helsinki.

### Statistics

Prior to analysis, the genotype distributions of all three samples (case-control GWA sample, population based GWA sample, and the family sample) were tested for deviations from Hardy-Weinberg equilibrium using an exact two-sided test [9]. The association between increased BMI and *CTNNBL1 *polymorphisms in the KORA cohort was analysed using linear regression analysis adjusted for age and sex while logistic regression was used for data from the case-control GWA. In both cases we used an additive model for the risk allele as described in [6]. In our family-based study we tested for overtransmission of the rs6013029 "T" allele – reported in the original study as being the risk allele – to affected offspring with the Pedigree Disequilibrium Test (PDT-sum) [10] and generated a genotype relative risk estimates using conditional logistic regression.

Power calculations based on the effect of genetic variants in rs6013029 were performed for the case-control and the cohort using the program QUANTO Version 1.2.3  and for the family-based sample using TDT Power Calculator 1.2.1 . For these calculations we assumed a minor allele frequency = 0.05 and genetic effect size of OR = 1.42 as estimated in [6] for the tests which used the case-control and family setting, while a true genetic effect of β = 0.1 (increase in mean BMI with each additional risk allele) was chosen for the cohort. In either case α = 0.05 (one-sided) was chosen.

## Results and discussion

We analysed the data of both GWA studies on the SNPs previously reported in [6] of the *CTNNBL1 *gene. There was no indication of a deviation from Hardy-Weinberg equilibrium at any of these markers in either GWA sample or among the founders in the families based on the exact test described above (all p-values > 0.05). Furthermore, there was no evidence for an association of any of the SNPs in the *CTNNBL1 *gene with obesity in our data (Table 1). The strongest signal in the original report (rs6013029) achieved a two-sided p-value of 0.53 in our case-control GWA with an estimated odds ratio (OR) of 0.88 (95% confidence interval (CI) 0.60 – 1.30) for the risk-allele T. Even though there is some overlap in the confidence intervals when comparing our result to the results of the original report's French case-control validation sample with 1.42 (95% CI 1.14 – 1.77) the point estimators indicate different directions of the T-allele effect. Combined with the absence of an observed association of this marker with BMI in the KORA cohort a false positive initial observation is the most likely explanation (Table 1).

**Table 1:** Results of the best SNPs in *CTNNBL1 *as previously described [6] in two independent GWAs.

**Case-control^1^**									**KORA cohort^2^**			
	
**SNP**	**Alleles^4^**	**MAF^5 ^cases/controls**	**Genotype distribution cases (%)/controls (%)**			**Odds ratio (95% CI)**	**Two-sided *p*-value**	**One-sided *p*-value**	**MAF**	**β (95% CI)**	**Two-sided *p*-value**	**One-sided *p*-value**
	
**rs6013029^3^**	**G**/T	0.057/ 0.064	432(88.7)/ 387(87.6)	54(11.1)/ 53(12.0)	1(0.2)/ 2(0.4)	0.884 (0.603 – 1.296)	0.528	0.736	0.051	0.1013 (-0.522 – 0.724)	0.750	0.375
**rs16986921**	**C**/T	0.056/ 0.062	433(88.9)/ 388(88.0)	53(10.9)/ 51(11.6)	1(0.2)/ 2(0.4)	0.899 (0.611 – 1.324)	0.591	0.705	0.052	0.1585 (-0.463 – 0.780)	0.617	0.309
**rs6020712**	**G**/A	0.056/ 0.064	433(88.9)/ 387(87.6)	53(10.9)/ 53(12.0)	1(0.2)/ 2(0.4)	0.867 (0.591 – 1.273)	0.467	0.766	0.052	0.1052 (-0.515 – 0.725)	0.740	0.370
**rs6020846**	**A**/G	0.066/ 0.070	424(87.1)/ 383(86.7)	62(12.7)/ 56(12.7)	1(0.2)/ 3(0.7)	0.932126 (0.649 – 1.340)	0.704	0.648	0.060	0.0531 (-0.529 – 0.635)	0.858	0.429
**rs6020395**	**G**/C	0.070/ 0.075	418(86.2)/ 376(85.6)	66(13.6)/ 60(13.7)	1(0.2)/ 3(0.7)	0.927 (0.650 – 1.320)	0.673	0.664	0.064	0.1279 (-0.444 – 0.700)	0.661	0.331
**rs16986890**	**A**/G	NA^6^							0.061	-0.1227 (-0.682 – 0.436)	0.667	0.666
**rs6096781**	**T**/C	0.063/ 0.055	427(87.7)/ 396(89.6)	59(12.1)/ 43(9.7)	1(0.2)/ 3(0.7)	1.136 (0.773 – 1.670)	0.515	0.257	0.053	0.0956 (-0.517 – 0.708)	0.760	0.380
**rs6020339**	**T**/C	0.372/ 0.070	197(40.5)/ 383 (86.6)	216(44.4)/ 56/(12.7)	73(15.0)/ 3 (0.7)	0.962 (0.651 – 1.346)	0.678	0.661	0.380	-0.0032 (-0.447 – 0.715)	0.982	0.509

^1 ^described in Hinney et al., 2007 [7]

^2 ^described in Wichmann et al., 2008 [8]

^3 ^best SNP in the original report [6]

^4 ^minor allele in bold

^5^MAF = minor allele frequency

^6^NA = Not available in the Affymetrix^® ^Genome-Wide Human SNP Array 5.

As rs6013029 was the main initial finding [6], we nevertheless decided to genotype this variant in 775 independent families ascertained for at least one obese offspring. We detected no evidence for an overtransmission of the T-allele – risk allele in the original study – to the obese offspring (two-sided p = 0.50), and an effect size estimate based on this sample of genotype relative risk (GRR) = 0.933 (95% CI 0.717 – 1.214) failed to exclude unity as well. The other *CTNNBL1 *SNPs previously described in [6] are displayed in Table 1. In addition, we also tried to explore the unvalidated results of SNPs from three additional genes (*FLJ42133*: rs7363432 and rs6095722; *SH3PXD2B*: rs13356223, rs10077897 and rs13436547; *SLIT3*: rs17734503 and rs12654448) which were also reported to be associated with increased fat mass (*FLJ42133*) or increased BMI (*SH3PXD2B *and *SLIT3*), respectively (Table 2). Once again, no evidence for an association in either of our GWAs was detected. In view of the requirement that replication studies need to be adequately powered, we assessed the power of each or our three samples based on the parameters listed above. For the given samples sizes our family-based replication study had a power > 80%, the cohort had a very limited power of about 10% while the case-control GWA had a slightly larger power of about 54%. If the initially reported values overestimate the true genetic effect, which is presumably quite often the case [11], our data nevertheless contribute to a more precise idea of the impact of *CTNNBL1 *variants on obesity.

**Table 2:** Results of the best SNPs in *SH3PXD2B, SLIT3 *and *FLJ42133 *as previously described [6] in two independent GWAs.

**Case-control^1^**										**KORA cohort^2^**			
	
**Gene**	**SNP**	**Alleles^3^**	**MAF^4 ^cases/controls**	**Genotype distribution cases (%)/controls (%)**			**Odds ratio (95% CI)**	**Two-sided *p*-value**	**One-sided *p*-value**	**MAF**	**β (95% CI)**	**Two-sided *p*-value**	**One-sided *p*-value**
	
**SH3PXD2B**	**rs13356223**	T/**C**	0.033/ 0.032	454 (93.4)/ 410 (93.8)	32(6.6)/ 26 (6.0)	0/ 1 (0.2)	1.029 (0.614 – 1.722)	0.915	0.457	0.041	-0.549 (-1.235 – 0.136)	0.116	0.942
	**rs10077897**	G/**A**	0.040/ 0.036	447 (92.0)/ 411 (93.0)	39 (8.0)/ 30 (6.8)	0/ 1 (0.2)	1.114 (0.690 – 1.799)	0.658	0.329	0.046	-0.430 (-1.086 – 0.225)	0.198	0.901
	**rs13436547**	G/**A**	0.033/ 0.032	455 (93.4)/ 415 (93.9)	32 (6.6)/ 26 (5.9)	0/ 1 (0.2)	1.038 (0.620 – 1.739)	0.886	0.443	0.041	-0.486 (-1.172 – 0.201)	0.165	0.917
**SLIT3**	**rs17734503**	A/**G**	0.082/ 0.072	409 (84.0)/ 380 (86.0)	76 (15.6)/ 60 (13.6)	2 (0.4)/ 2 (0.4)	1.150 (0.814 – 1.626)	0.428	0.214	0.086	0.358 (-0.109 – 0.825)	0.133	0.066
	**rs12654448**	C/**T**	0.079/ 0.071	410 (84.4)/ 381 (86.2)	75 (15.4)/ 59 (13.3)	1 (0.2)/ 2 (0.5)	1.126 (0.791 – 1.602)	0.510	0.255	0.088	0.125 (-0.352 – 0.601)	0.608	0.304
**FLJ42133**	**rs7363432**	A/**G**	0.066/ 0.070	424 (87.1)/ 383 (86.6)	62 (12.7)/ 56/(12.7)	1 (0.2)/ 3 (0.7)	0.932 (0.649 – 1.340)	0.704	0.648	0.057	0.234 (-0.369 – 0.837)	0.447	0.223
	**rs6095722**	A/**G**	0.066/ 0.070	422 (87.0)/ 383 (86.6)	62 (12.8)/ 56/(12.7)	1 (0.2)/ 3 (0.7)	0.936 (0.651 – 1.346)	0.722	0.639	0.060	0.134 (-0.447 – 0.715)	0.651	0.325

^1 ^described in Hinney et al., 2007 [7]

^2 ^described in Wichmann et al., 2008 [8]

^3 ^minor allele in bold

^4^MAF = minor allele frequency

In sum, our results underline the importance of replication of GWA results in independent samples even though independent validations may have been reported within the same initial study. While replication of association with obesity of intron 1 variants in *FTO *has been demonstrated robustly in almost all subsequent studies comprising obese adults and children [7,12-15], the study by Liu et al. [6] was an exception as none of the intron 1 *FTO *SNPs showed evidence for a body weight-related association. Interestingly, however, the study did find some evidence for an association of variants in *INSIG2 *with obesity [5,16-19]. Both examples underline the difficulties that arise when trying to validate, confirm and replicate associations with such complex traits as obesity. Our failure to replicate the initial findings [6] also does not appear to be a result of population stratification. All recruitment was done in Germany for which population stratification effects have shown to be of minor importance [20].

Another possible explanation for a lack of replication is that our results are mainly based on data for children and adolescents which are different from [6] where only adults were investigated. Again the example of *FTO *[6] highlights how validated associations found in adults with obesity may also be present in children with extreme obesity [3,21]. Recently, two independent studies comprising more than 32,000 [22] and 14,000 [23] individuals also did not find significant association of the *CTNNBL1 *variant rs6013029 and obesity. Our study is a replication and validation attempt with sufficient combined power to independently replicate an initial finding [6], while also providing some evidence to support the decision not to follow-up variants that did not "survive" a validation within the same initial report [6]. Although we were not able to replicate the original findings, our data may be useful for a meta-analytical assessment of the association of *CTNNBL1 *variants and obesity. A retrospective look at the conflicting reports on *INSIG2 *and the recent reports on *CTNNBL1 *suggests that research on mediating and moderating variables to more comprehensively assess phenotype-genotype relationships is urgently needed.

## Conclusion

We did not detect confirmation of association of variants in *CTNNBL1 *with obesity in a population of Central European ancestry. Further studies have to be performed to validate or not the initial findings about the association of *CTNNBL1 *variants and obesity.

## Competing interests

The authors declare that they have no competing interests.

## Authors' contributions

CIGV participated of the study design, perform the genotyping and drafted the manuscript; BG performed statistical analysis and drafted the manuscript; AS participated of the study design, performed secondary statistical analyses and drafted the manuscript; TM, SF, HG, IMH, TI, HEW and HS participated of the study design; JH and AH conceived the study, participated in its design and coordination and drafted the manuscript. All authors read and approved the final manuscript.

## Pre-publication history

The pre-publication history for this paper can be accessed here:


